# Synthesis and pharmacological properties of coumarin‐chalcones

**DOI:** 10.1016/j.mex.2023.102488

**Published:** 2023-11-15

**Authors:** Donia Bensalah, Nasser Amri, Yousef E. Mukhrish, Waleed S. Koko, Naceur Hamdi

**Affiliations:** a Research Laboratory of Environmental Sciences and Technologies (LR16ES09), Higher Institute of Environmental Sciences and Technology, University of Carthage, Hammam-Lif, Tunisia; b Department of Chemistry, Faculty of Science, Jazan University, P.O. Box 2097, Jazan 45142; Saudi Arabia; c Department of Science Laboratories, College of Science and Arts, Qassim University, Ar Rass 51921, Saudi Arabia

**Keywords:** Coumarin, Antibacterial, Antioxidant, *Anti-inflammatory* activity and *antiproliferative* activity, Coumarin chalcones hybrids, Synthesis of coumarin -bonded chalcone derivatives via two steps reaction

## Abstract

New chalcones (2a-e) were prepared by Claisen-Schmidt condensation from 3-acetyl-4-hydroxycoumarin, which was used as a key intermediate in this synthesis. However, we can easily obtain compounds (3a-e) by refluxing chalcone (2a-e) with 4-hydroxycoumarin in the presence of ammonium acetate and glacial acetic acid. Multinuclear NMR (^1^H and ^13^C), IR and elemental analysis characterized the structure of the final compound. The antibacterial activity of the obtained products against various bacterial strains was tested in vitro. The antioxidant properties of the same synthesized compounds were also studied using DPPH (2,2-diphenyl-1-trinitrophenylhydrazine) and hydroxyl radical scavenging tests. Furthemore a study was conducted to highlight the nature of the effects produced by screening 2a-e and 3a-e products on colon cancer cell lines (HCT-116) and hepatocellular carcinoma cell lines (HepG-2). Good cytotoxic activity against standard vinblastine was observed for compound 3a.

• 3-acetyl-4-hydroxycoumarin as a simple coumarinic ketone was modified to coumarins-bonded chalcones.
• Modification was performed through two steps reaction.
• Final products exhibited free radical scavenging activity and Good cytotoxic

## Graphical abstract

**Figure:**
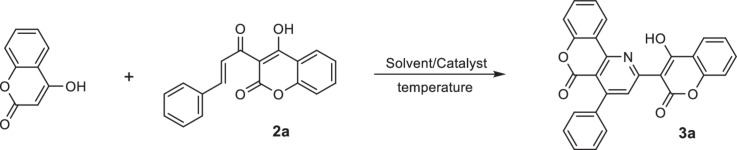

Method name: Synthesis of coumarins-bonded chalcone derivatives via two reaction: aldol condensation, and then copper-mediated azide-alkyne cyclization

Specifications table

**Table:** 

Subject area:	Chemistry
More specific subject area:	Organic Chemistry
Name of your method:	Synthesis of coumarin -bonded chalcone derivatives via two steps reaction
Name and reference of original method:	Novel coumarin-chalcone derivatives: Synthesis, characterization, antioxidant, cyclic voltammetry, molecular modelling and biological evaluation studies as acetylcholinesterase, α-glycosidase, and carbonic anhydrase inhibitors. Chemico-Biological Interactions 383 (2023) 110655.
Resource availability:	The investigation was conducted in the Research Laboratory of Environmental Sciences and Technologies (LR16ES09), Higher Institute of Environmental Sciences and Technology, University of Carthage, Hammam-Lif, Tunisia
The reagents and chemicals used were purchased from commercial suppliers such as Merck, Sigma-Aldrich. The instruments used were FTIR, ^1^H and ^13^C NMR, and HRMS.	

## Method details

Chalcones are excellent compounds with a α,-unsaturated ketone functionality between the two aromatic rings. They are flavonoid precursors that have been extensively studied as important structural elements in drug development [1]. Recent studies have reported many pharmacological properties of chalcones and their derivatives, including antispasmodic, anti-inflammatory, antimalarial, anticancer, antioxidant, antibacterial, antiproliferative, and xanthine oxidase inhibitory effects [[2], [3]]. The pharmacological importance of naturally derived compounds motivates researchers to obtain hybrid molecules with different structures and biological effects. [4], [5], [6], [7]. Coumarins and chalcones are two important classes of natural products that have attracted great interest due to their excellent and diverse biological activities [8], [9], [10].

**Fig. 1:**
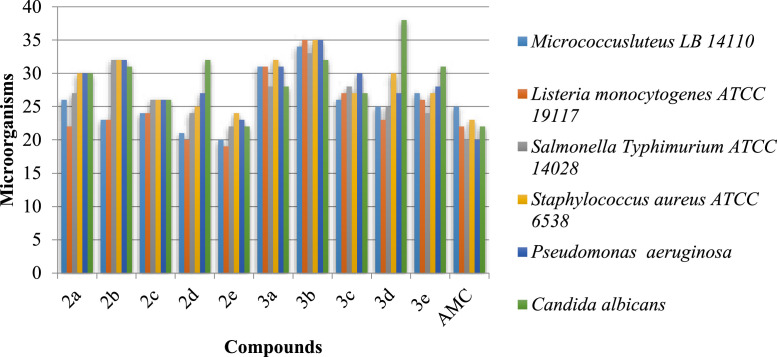
Zone of bacterial inhibition measured in mm of the synthesized chalcones 2a-e and compounds 3a-e.

Structurally, coumarins represent a broad group of vital lactones containing a benzene ring fused to a 2-pyrone structure (Fig. 1) and are widely distributed in plants. Interest in natural and synthetic molecules containing the coumarin backbone continues to grow due to their use as medicinal agents because of their exceptional therapeutic potential as anticancer, anticoagulant, antitubercular, antimicrobial, anti-inflammatory, anti-HIV, analgesic, anticonvulsant, antiplatelet, antifungal, antiviral, antibacterial, and antimalarial activities [11]. Chalcone (1,3-diaryl-2-propen-1-one, Fig. 1), is one of the bioactive secondary metabolites belonging to the favonoid family and is of considerable interest for a wide range of biological properties such as anticancer, antifungal, *anti-inflammatory, analgesic, antibacterial, antimalarial* and antioxidant activities [[8], [12]]. The multifunctionality of coumarin and chalcone has shown that they are ideal building blocks to create a hybrid scaffold with interesting pharmacological properties. This explains and proves that the idea of a hybrid of coumarin and chalcone possessing diverse and impressive pharmacological activities is well assured as they have shown anticancer, antimicrobial, antimalarial, antioxidant and a*ntitubercular* activities [13], [14], [15], [16], [17].

On the other hand, coumarin (2H-1-benzopyran-2-one) of natural or synthetic origin is widely distributed in plants and has important pharmacological [18], enzyme inhibitory [[19], [20]] and anti-tumor effects [[21], [22]] and antiviral [[23], [24]] activity as well as anti-inflammatory [[25], [26]] and antioxidant [27], [28], [29], [30], [31], [32], [33], [34], [35], [36], [37], [38], [39], [40], [41], [42], [43], [44], [45] properties. Due to the advantages of heterocyclic organic compounds, many researchers have focused their research on the synthesis of heterocyclic platform chalcone derivatives. The aim of our method is the synthesis of coumarin-conjugated chalcones from simple 3-acetyl-4-hydroxycoumarin.

**Scheme 1:**
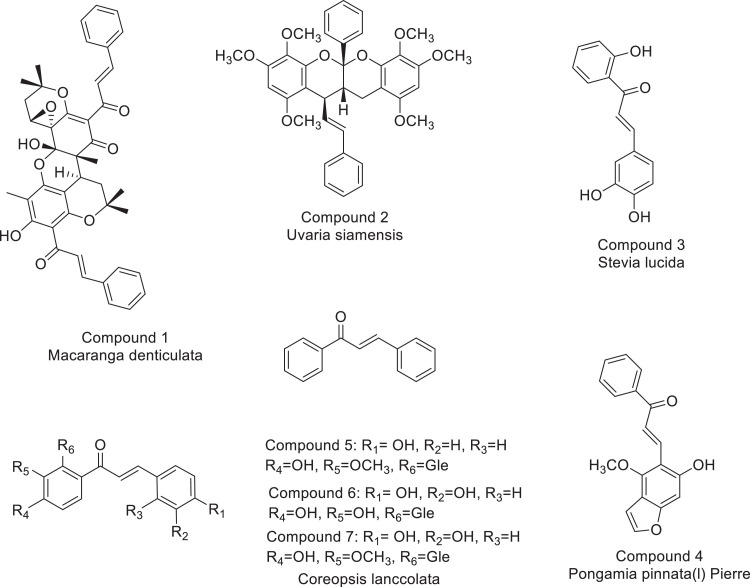
Basic structure of chalcone (middle) and some naturally occurring chalcones.

To do it 3-Acetyl-4-hydroxycoumarin (1) was used as starting material. 3-Acetyl-4-hydroxycoumarin (0.005 mol), aromatic aldehyde (0.005 mol) and chloroform (10 mL) were added to a 50 mL round bottom flask. Piperidine (0.005 mol) was added to the reaction mixture and refluxed for 1.5 h. After the chloroform was evaporated, the residue was recrystallized from methanol to obtain the corresponding (E) 4-hydroxy-3(3′-arylacryloyl)-2H-benzopyran-2-one 2. Aldol condensation used in the synthesis of chalcones (similar compounds). (Based on our study) It has been reported by Sharma et al. implement. (2014) using aqueous methanol-sodium hydroxide solution in alkaline medium. However, in our experiments, it was difficult to perform aldol condensation under such conditions (Scheme 1).

The structures of the obtained compounds are fully characterized by different spectroscopic methods, including multinuclear NMR (^1^H and ^13^C), FT-IR and elemental analysis.

**Scheme 2:**
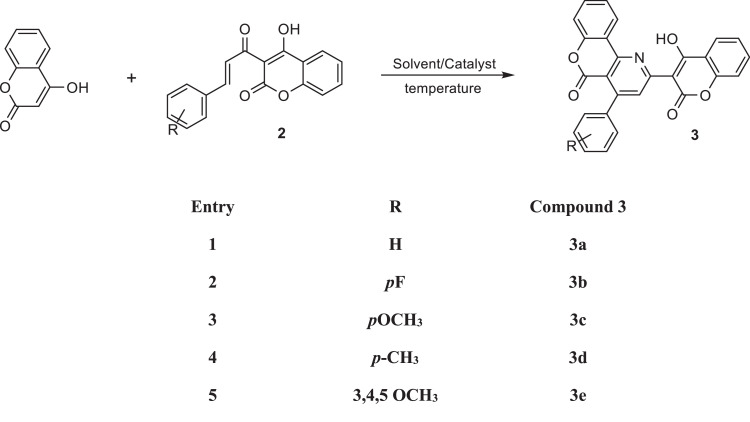
Protocol synthesis of compounds 3a-3e.

The proton NMR spectrum of 3-acetyl-4-hydroxycoumarin shows an OH signal at 17.72 ppm. This occurrence in this group at very low fields can be explained by the formation of intermolecular hydrogen bonds [[46], [47]]. In the second step, ammonium acetate (0.05 mol) was added to a solution of 2 (0.005 mol) in glacial acetic acid (15 ml) with stirring at room temperature. A solution of 4-hydroxycoumarin (0.005 mol) in glacial acetic acid (15 ml) was then added while stirring at room temperature for the next 15 min. The reaction mixture was then refluxed in an oil bath at 140 °C for 8 h and then stirred at room temperature for a further 45 min. It was then allowed to reach room temperature and poured into ice water (75 mL). The resulting mass was than extracted with chloroform (3 × 30 ml). The combined chloroform extracts were washed first with water and then with 10 % sodium bicarbonate solution (3 × 20 mL) (Scheme 2).

The proposed structures of the synthesized coumarin chalcones 3 were in perfect agreement with the spectral data and elemental analysis. FT-IR spectra of 2a-e recorded in the KBr disk showed absorption bands ν = 979 cm^−1^ corresponding to the C = Ctrans group, ν = 1659 cm^−1^ characterizing the carbonyl group, ν = 1598 cm^−1^ corresponding to the C = Carom group, while Csp2-H stretching vibrations in the aromatic region ν = 3057 cm-1. The trans-olefinic protons Ha and Hb appear as orthocoupled doublets in the ^1^H NMR spectrum at about 8.28 (J = 15.6 Hz) and 8.33 (J = 15.9 Hz), respectively. The methoxyl protons resonate at 3.80 ppm. As for the aromatic protons, they appeared between 6.81 and 8.33 ppm. Similarly, the ^13^C-NMR spectrum of product 2c showed a signal at 55.4 ppm attributed to the methoxyl group carbon, characteristic signals at δ = 114.4 ppm, δ = 147.4 ppm, and δ = 162.4 ppm corresponding to the C_3_, C_2_, and CO carbons, respectively. The aromatic region shows signals at 116.8 and 147.4 ppm. Given the variety of substituents grafted to the aromatic ring of the 2a-e precursors, a wide range of 2-(4-hydroxy-2-oxochroman-3-yl)-4-(p-alkyl)-5H-chromeno[4,3-b]pyridin-5-ones 3a-e can be readily obtained. The reaction was then studied under different conditions (solvents, reaction time, temperature and catalysts).

To study the catalytic activity, different catalysts including TFA, HCOOH, ZnO and glacial acetic acid were selected. As a model, the condensation reaction between chalcone 2a and 4-hydroxycoumarin was studied by operating under standard conditions, at different temperatures and with each catalyst present separately. Table 2 shows the results obtained.

TFA, HCOOH, CH_3_COONH_4_, and ZnO were tested for their catalytic effects (Table 1, entries 10–15). CH_3_COONH_4_ in glacial acetic acid was found to be the most effective catalyst for this reaction. Different concentrations of this catalyst were then evaluated and it was initially found that 5 mol% of the catalyst produced 65 % of the yield. Increasing the amount of catalyst to 10, 15, and 20 mol% increased the yield to 95 %, 92 %, and 85 %, respectively. The results show that beyond 20 mol%, the yield of the reaction does not improve significantly, so the 10 mol% value of the catalyst was chosen as the maximum amount of catalyst for the reaction. As for the reaction time, 8 h was sufficient to complete the reaction (Table 1, entry 15). From the above, the use of ammonium acetate (10 mol%) in glacial acetic acid for 8 h would give the best conditions for this reaction. Keeping the above conditions, the reaction is carried out with different series of aromatic aldehydes to study the limitations of this methodology. Table 3 summarizes the results obtained. Condensation with 4-hydroxycoumarin was successful for aromatic aldehydes that closed both electron-withdrawing and electron-donating functional groups (Table 3, entries 2-5).

**Table 1:** Synthesized of (E) 4-hydroxy-3(3’-arylacryloyl)- 2H-chromene-2-one 2.


Entry	R	Compound 2	Yield^a^^,^^b^ (%)
1	H	2a	90
2	*p*F	2b	85
3	*p*OCH_3_	2c	92
4	*p*-CH_3_	2d	95
5	3,4,5 OCH_3_	2e	83

a All reactions were carried with 3-acety-4-hydroxycoumarin (0.005 mol), arylaldehyde (0.005 mol), piperidine(0.005 mol) and 10mL (CHCl_3_).

b Yield of the isolated product.

**Table 2:** Optimization of the reaction conditions using different solvents.


Entry	Catalysts (mmol %)	Solvent	Time (h)	Temperature (°C)	Yield^a^^,^^b^^,^^c^ (%)
1	No	EtOH	24	78	-
2	No	CH_2_Cl_2_	24	40	-
3	No	Toluene	24	110	-
4	No	CH_3_CN	24	82	-
5	No	THF	24	66	-
6	No	Glacial AcOH	24	119	-
7	TFA (10)	Glacial AcOH	24	119	45
8	HCOOH (10)	Glacial AcOH	24	119	42
9	ZnO(10)	Glacial AcOH	24	119	Trace
10	CH_3_COONH_4_ (5)	Glacial AcOH	24	119	65
11	CH_3_COONH_4_ (10)	Glacial AcOH	24	119	95
12	CH_3_COONH_4_ (15)	Glacial AcOH	24	119	92
13	CH_3_COONH_4_ (20)	Glacial AcOH	24	119	85
14	CH_3_COONH_4_ (10)	Glacial AcOH	12	119	85
15	CH_3_COONH_4_ (10)	Glacial AcOH	8	119	95
16	CH_3_COONH_4_ (10)	Glacial AcOH	6	119	75

a All reactions were carried with 3-acety-4-hydroxycoumarin (0.005 mol), arylaldehyde (0.005 mol), piperidine(0.005 mol) and 10 mL (CHCl_2_).

b Yield of the isolated product.

c The reaction failed to provide a product. Glacial AcOH.

**Table 3:** Chemical yields and physical properties of coumarins derivatives 3.

Entry	R	Compound^a^ 3	Yield^b^ (%)
1	H	3a	95
2	*p*F	3b	85
3	*p*OCH_3_	3c	80
4	*p*-CH_3_	3d	75
5	3,4,5 OCH_3_	3e	85

a All reactions were carried with 3-acety-4-hydroxycoumarin (0.005 mol), arylaldehyde (0.005 mol), piperidine(0.005 mol) and 10mL (CHCl_2_).

b Yield of the isolated product.

All synthesized compounds 3a-e were characterized in solution by NMR (^1^H and ^13^C), IR spectroscopy and elemental analysis. As an example, the IR spectrum of compound 3c shows a very strong band at 1683 cm^−1^ related to the carbonyl stretching vibration (C=O) of the -lactone ring of the coumarin nucleus. The C-O and C=N stretching vibrations appear at 1714 and 1608 cm^−1^, respectively. The stretching vibrations of the aromatic C-H were detected between 3093 and 3144 cm^−1^. The aromatic proton signals in the spectra of compound 3c were found as a multiplet between 6.50 and 8.50 ppm. A singlet at δ 3.57 ppm was assigned to the methoxy protons. In the ^13^C-NMR spectra, all compounds showed signals at the expected values. The two carbonyl carbon signals of the -lactone appeared between δ 160.0 and δ 181.0 ppm, while the aromatic carbon signals were seen between 95.0 and 176.0 ppm. Quaternary and tertiary carbons were distinguished by DEPT-135.

**Scheme 3:**
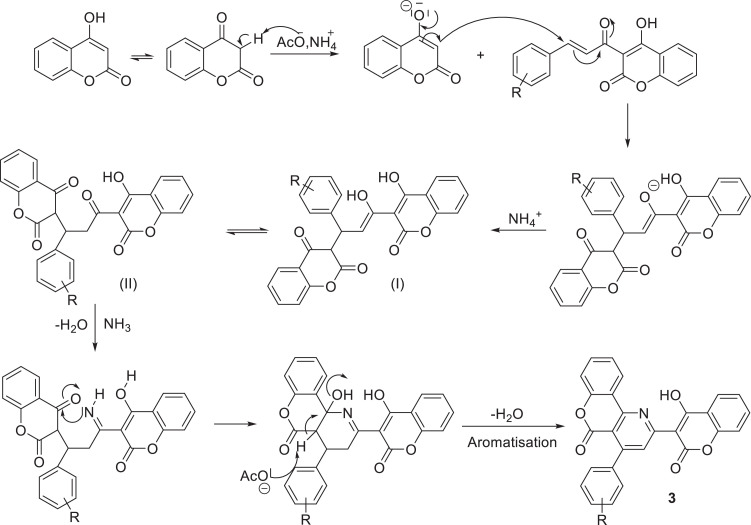
Plausible reaction mechanism for the formation of compounds 3.

Mechanistically, the reaction begins with the deprotonation of 4-hydroxycoumarin with acetate ions. Then the obtained enolate attacks the double bond of chalcone 2, leading to equilibrium between the enol (I) and its keto form (II) after successive acid-base reactions. The latter reacts with the ammonia formed to form an imine, which is cyclized by nucleophilic attack of its nitrogen on the coumarin ketone, which after aromatization leads to the formation of a pyridine ring and thus the final product 3 (Scheme 3).

The obtained products were then subject to the following biological activities.

### Biological activities

#### Antibacterial and antifungal activities

**Fig. 2:**
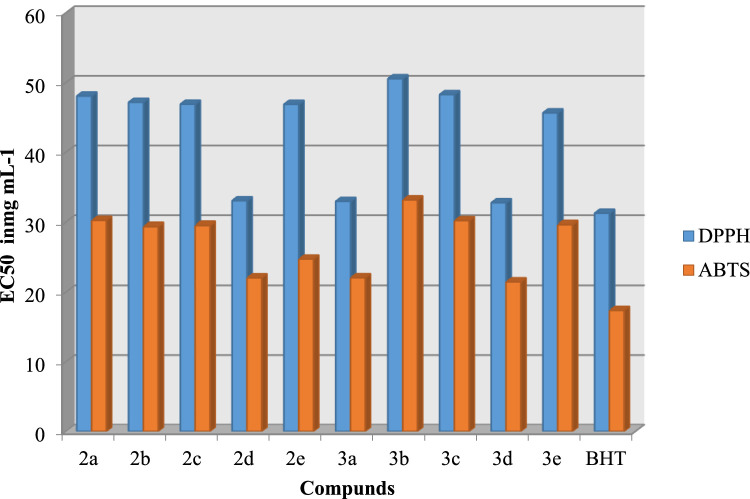
Antioxydant activity of the synthesized compounds 2a-e et 3a-e assessed by DPPH and ABTS techniques and expressed as IC_50_ in mg mL^−1^. The BHT was used as control.

The in vitro antibacterial and antifungal activities of the synthesized compounds 2a-e and 3a-e against different Gram-positive, Gram-negative and fungal species were evaluated. The results obtained are shown in Figs. 1 and 2. The compounds 3a-e showed a wide range of antibacterial activity against all the bacterial strains tested (0.125-0.624 mg/mL). In addition, compound 3b is active against *Listeria monocytogenes* ATCC 19117, *Salmonella Typhimurium* ATCC 14028, and *Micrococcus luteus* LB14110 at concentrations of 0.31, 0.3134, and 0.421 mg/mL, respectively, having a fluorine substituent at the para position of the aryl ring. Compound 3d shows effective inhibitory activity in terms of MIC = 0.2124 mg/mL against *Listeria monocytogenes* ATCC 19117, having a methyl substituent donating electrons at the para position of an aryl ring. A moderate activity (MIC = 1.34 mg/mL, 24 mm zone of inhibition) against *Salmonella Typhimurium* ATCC 14028 was observed for compound 3e with OCH_3_ substituent at the 3,4,5-position of the aryl ring.

The Minimum inhibitory concentration (MIC) against the above organisms was determined by the method of broth dilutions [48]. The results are given in Table 4.

**Table 4:** Minimal bacterial inhibitory concentration measured in mg/mL of compounds 3.

Microorganism indicator	Compounds	MIC (mg/ml)
*Listeria monocytogenes* ATCC 19117	3a	0.52
	3b	0.31
	3c	0.12
	3d	0.21
	3e	0.42
	Ampicillin	0.03
*Salmonella* Typhimurium ATCC 14028	3a	1.26
	3b	0.31
	3c	1.16
	3d	0.21
	3e	1.34
	Ampicillin	0.03
*Micrococcus luteus* *LB14110*	3a	0.62
	3b	0.42
	3c	0.32
	3d	0.22
	3e	0.12
	Ampicillin	0.03

We found that all compounds 2 and 3 are very active. They were compared with ampicillin which was used as a control antibiotic against the strains. Compound 3e with an MIC of 0.125 mg/mL showed good activity against Micrococcus luteus compared to ampicillin.

#### Antioxidant activity

The synthesized compounds 3a-e and 2a-e were tested for their in vitro antioxidant activity by means of DPPH radical scavenging assay, hydroxyl radical scavenging assay and superoxide radical scavenging assay. Fig. 2 summarizes the IC_50_ values of the standards and test samples. Compounds 3c, 3d showed antioxidant activities at 14 µM concentration in all three antioxidant assays. However, good antioxidant activity (12 - 13 µM) was observed for compounds 3b and 3e.

#### Anti‐inflammatory activity

**Fig. 3:**
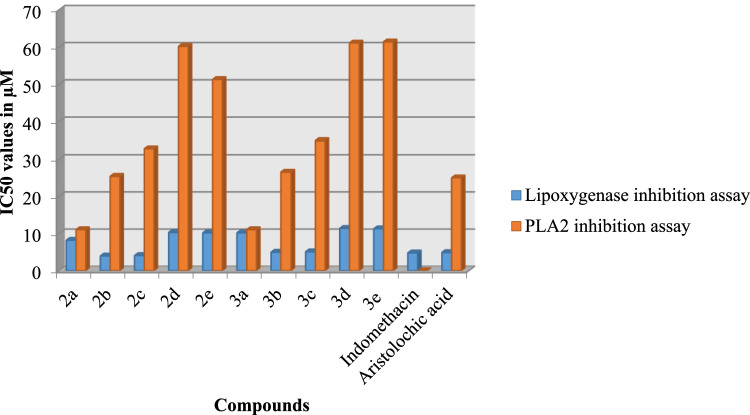
IC_50_ values of the compounds 2a-e and their compounds 3a-e for anti-inflammatory activity.

Lipoxygenase inhibition and phospholipase A_2_ (PLA_2_) inhibition assays were then used to test the synthetic compounds 3a-e in accordance with our objective. The IC_50_ values for the standards and test samples in both assays are presented in Fig. 3. The synthesized compounds 3b and 2c showed strong anti-inflammatory activity in both the PLA_2_ inhibition assay (26.5–25.4 M) and the lipoxygenase inhibition assay (5.0–4.1 M).

#### Antiproliferative activity

**Fig. 4:**
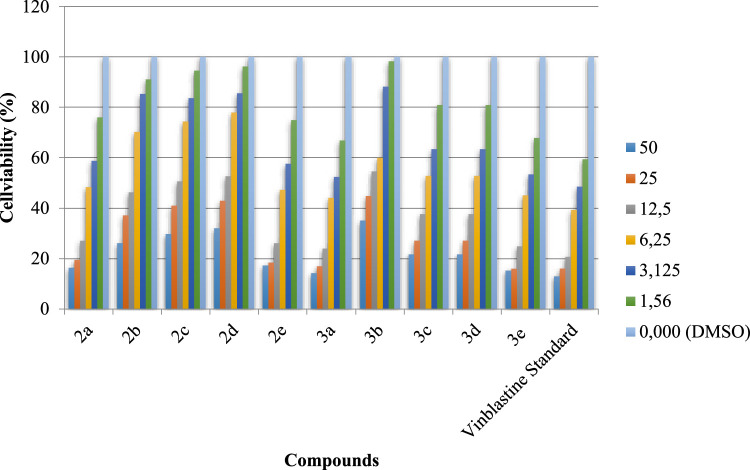
Evaluation of cytotoxicity of the synthesized compounds (2a-2e) and (3a-3e) against colon carcinoma cells (HCT-116).

**Fig. 5:**
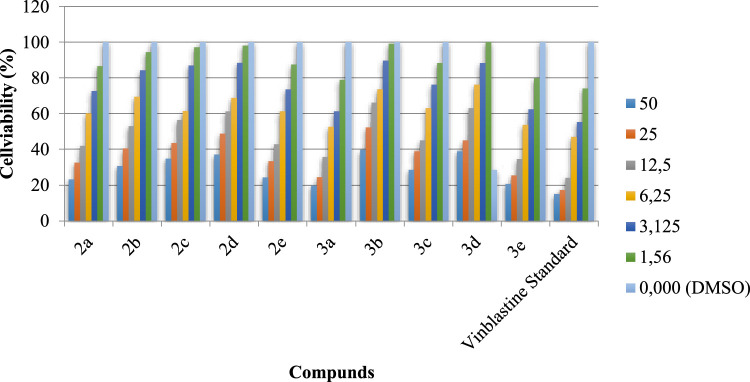
Evaluation of cytotoxicity of the synthesized compounds (2a-2e) and (3a-3e) against hepatocellular carcinoma cells lines (HepG-2).

**Fig. 6:**
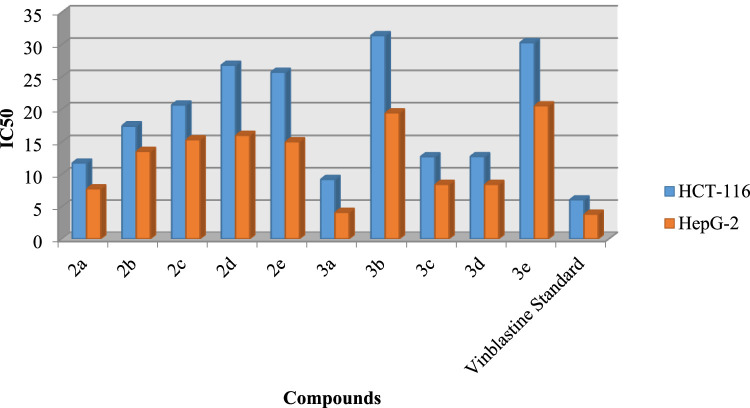
IC_50_ of the synthesized compound (2a-2e) and (3a-3e) on colon carcinoma cells (HCT-116) and hepatocellular carcinoma cells lines (HepG-2).

The synthesized compounds (2a-2e) and (3a-3e) were tested for potential cytotoxicity against colon cancer (HCT-116) and hepatocellular carcinoma (HepG) using the methods of Mossman [49], Gangadevi and Muthumary [50] and vinblastine as a standard cell line. IC50 is the concentration required to account for 50 % of cell viability. Screening of selected compounds against human colon cancer cell lines and hepatocellular carcinoma cell lines showed that compound 3a exhibited IC50 values (14.27 and 30.79 μg) in both human colon cancer cell lines and hepatocellular carcinoma cell lines. These values are roughly equivalent to the standard drug vinblastine (3.83 and 6.05 µg). Fig. 6 At the same time, compounds 2a, 2b, and 3c showed significant cytotoxic activity, with IC_50_ values in the range of (14.27–26.81) µg in human colon cancer cell lines and (23.34-30.79) µg in hepatocellular carcinoma cell lines. Additionally, compounds 2c, 2d, 3b were weakly cytotoxic with IC_50_ values ranging from (29.78-35.18) µg in human colon cancer and (34.92–37.26) µg in hepatocellular carcinoma cell lines. The IC50 values also indicate that the increased toxicity in the hepatocellular carcinoma cell lines requires the use of higher doses as compared to the human colon cancer cell lines (Figs. 4 and 5).

## Conclusion

In summary, we have developed an easy-to-use catalytic system that can efficiently promote the conversion of 3-acetyl-4-hydroxycoumarin to chalcone. Compounds 3a–e were then obtained by reacting compound 2 with 4-hydroxycoumarin in ACOH. Synthetic compounds 2a-e and 3a-e were evaluated against four indicator microorganisms, including the fungus Candida albicans and three bacteria Listeria monocytogenes, Staphylococcus aureus, and Salmonella typhimurium. Compound 3e showed broad-spectrum inhibitory activity against the four tested microorganisms and was the most potent compound. In addition, the MIC value of compound 3e against *Listeria* was 0.426 g mL-1. The synthesized compounds (2a-2e) and (3a-3e) were tested against colon cancer cell line (*HCT-116*) and hepatocellular carcinoma cell line (*HepG-2*).Only compound 3a showed significant cytotoxic activity, approximately equivalent to the activity of standard vinblastine. These results prompted us to propose inclusion of these compounds in antibiotic formulations to increase their sensitivity to cancer-stimulating and apoptosis-inducing antibiotics in human colon cancer and hepatocellular carcinoma.

## Experimental

### General information

All manipulations were performed using standard Schlenk techniques under an argon atmosphere. Chemicals were purchased from Sigma-Aldrich and used without further purification. All solvents were purified and dried using the MBRAUN SPS 800 solvent purification system. ^1^H NMR and ^13^C NMR spectra were recorded at 400 MHz and 100 MHz, respectively. Chemical shifts, δ, are reported in ppm relative to the internal standard TMS for both ^1^H and ^13^C NMR. Products were characterized by gas chromatography (GC). Quantitative GC analyses were performed using a GC-2010 Plus gas chromatograph (SHIMADZU). NMR studies were performed in high quality 5 mm NMR tubes. Signals are expressed in parts per million as δ downfield from tetramethylsilane (δ = 0.00) as internal standard. NMR multiplicities are abbreviated as follows: s = singlet, d = doublet, t = triplet, and m = multiplet signals. IR spectra were recorded on a 398 spectrophotometer. Elemental microanalysis was performed on an Elementar Vario El III Carlo Erba 1108 elemental analyzer, and the values obtained were within ±0.4 % of the theoretical values. Melting points were determined on a Kofler bench. The biological analysis was performed with reference to our previous work [51].

### General procedure for the synthesis of chalcone 2

Dissolve 3-acetyl-4-hydroxyxcoumarin (0.031 mol) and the substituted aromatic aldehyde (0.03 mol) in 30 ml of chloroform. A catalytic amount of piperidine (0.02 mol) was added to the reaction mixture and refluxed for 1.5 h. After distilling off the chloroform, the residue was recrystallized with methanol.

### 3-((2E)-3-(phenyl)prop-2-enoyl))-4-hydroxy-2(H)-chromen-2-one (2a)

Solid (yield 79 %), mp = 145°C, IR: m 3168 (–OH), 1622 (C=O), 1577 (C=C), 1028(s) (sym) (C–O–C); ^1^H NMR:(DMSO-d_6_, 300 MHz, tamb) δ ppm :7.2–8.5 (m, 10H, Ar–H, Hethy), 18.56 (s,1H, OH). ^13^C (DMSO-d_6_, 75MHz, tamb) δ ppm: 181.5 (CO); 166 (C_4_); 163.5 (C_2_);116.3–136.2 (Carom); 154.8 (=-Ph); 146.0 (=-CO).

C_18_H_12_0_4_ calc. C 73.97 H 4.10 O 21.92; found C 73.90 H 4.10 O 21.80.

### 3-((2E)-3-(4-fluorophenyl)prop-2-enoyl)-4-hydroxy-2(H)-chromen-2-one (2b)

Solid (yield 80 %), mp = 178°C, IR: m 3268 (–OH), 1625 (C=O), 1575 (C=C), 1025(s) (sym) (C–O–C); ^1^H NMR (DMSO-d_6_, 300MHz, tamb) δ ppm : 6.86–7.73 (m, 10H, Ar–H, Hethyl), 19.58 (s,1H, OH). ^13^C (DMSO-d_6_, 75 MHz, tamb) δ ppm: 192.2 (CO); 173.9 (C_4_); 172.2 (C_2_); 103.2–167.8 (Carom, Cethyl). C_18_H_11_0_4_ F calc. C 69.67 H 3.54 O 20.64 found C 69.70 H 3.60 O 20.70.

### 3-((2E)-3-(4-methoxyphenyl) prop-2-enoyl)-4-hydroxy-2(H)-chromen-2-one (2c)

Solid (yield 70 %), mp = 135°C, IR: m 3250 (–OH), 1620 (C=O), 1574 (C=C), 1025(s) (sym) (C–O–C); ^1^H NMR:(DMSO-d_6_, 300 MHz, tamb) δ ppm : 3.84 (s, 3H, OCH_3_), 6.91–8.33 (m, 10H, Ar–H, Hethy), 19.05 (s,1H, OH); ^13^C (DMSO-d_6_, 75MHz, tamb) δ ppm: 55.4 (OCH_3_); 162.4(CO); 147.4 (C=-Ph); 135.6 (=-CO);

114.4–134.5 (Carom). C_19_H_14_0_5_ calc. C 70.80 H 4.34 O 24.84 found C 70.70 H 4.40 O 24.80.

### 3-((2E)-3-(p-tolyl) prop-2-enoyl)-4-hydroxy -2(H)-chromen-2-one (2d)

Solid (yield 75 %), mp = 138°C, IR: m 3368 (–OH), 1627 (C=O), 1575 (C=C), 1030(s) (sym) (C–O–C); ^1^H NMR:(DMSO-d_6_, 300MHz, tamb) δ ppm : 2.3 (s, 3H CH_3_), 7.2–8.2 (m, 10H, Ar–H, Hethy),17.82 (s, 1H, OH); ^13^C (DMSO-d_6_, 75MHz, tamb) δ ppm: 30.0 (OCH_3_); 178.4(CO); 154.6 (C_4_); 135.6 (C_2_); 101.3–135.9 (Carom).C_19_H_14_0_4_ calc. C 74.50 H 4.57 O 20.91, found C 74.40 H 4.60 O 20.90.

### 3-((2E)-3-(3,4,5-trimethoxy-phenyl)prop-2-enoyl)-4-hydroxy-2(H)-chromen-2-one (2e)

Solid (yield 83 %), mp = 193°C, IR: m 3368 (–OH), 1716(s) (C=O), 1577 (C=C), 1018(s) (sym) (C–O–C); ^1^H NMR:(DMSO-d_6_, 300 MHz, tamb) δ ppm : 6.90–7.96 (m, 8H, Ar–H, Hethyl), 19.50(s, 1H, OH); ^13^C (DMSO-d_6_, 75 MHz, tamb) δ ppm: 191.6(CO); 59.7 (O-CH_3_); 59.8 (O–CH_3_). 60.0 (O–CH_3_) 103.47–153.3 (Carom); 164.8 (C_4_); 163.2 (C_2_); 102.8 (C_3_). C_21_H_18_0_7_ calc. C 65.96 H 4.71 O 29.31, found C 65.90 H 4.80 O 29.30.

### Synthesis of 2-(4-hydroxy-2-oxochroman-3-yl)-4-(p-alkyl)-5H-chromeno[4,3-b]pyridin-5-one 3

Ammonium acetate (0.05 mol) was added to a solution of 2 (0.005 mol) in glacial acetic acid (15 mL) with stirring at room temperature. A solution of 4-hydroxycoumarine (0.005 mol) in glacial acetic acid (15 mL) was then added while stirring at room temperature for the next 15 min. The reaction mixture was then refluxed in an oil bath at 140 °C for 8 h after stirring for another 45 min at room temperature. It was then allowed to reach room temperature before being poured into ice water (75 mL). The resulting gum mass was extracted with chloroform (3 × 30 mL). The combined chloroform extract was rinsed first with water and then with a 10 % sodium bicarbonate solution (3 × 20 mL).

### 2-(4-hydroxy-2-oxochroman-3-yl) -5H-chromeno[4,3-b]pyridin-5-one 3a

Solid (Yield 95 %), mp = 183°C; IR: m 3368 (–NH), 1716(s) (lactone C=O), 1577 (C=C), 1018(s) (sym) (C– O–C); ^1^H NMR (DMSO-d_6_, 300 MHz, tamb) δ ppm : 7.40 (s, 1H, Hethy), 7.96–8.20 (m, 4H, Ar–H), 13.2 (s, 1H, NH); ^13^C (DMSO-d_6_, 75MHz, tamb) δ ppm^:^ 158.2 (C_2_); 102.1 (C_3_); 149.2 (C_4_); 146.7 (C=N); 122.1–151.2 (Carom).

### 2-(4-hydroxy-2-oxochroman-3-yl) 4-(p-fluoro)-5H-chromeno[4,3-b]pyridin-5-one 3b

Solid (Yield 75 %), mp = 184°C; IR ν cm^–1^: 1720(s) (lactone C=O), 1578 (C=C), 1020(s) (sym) (C–O–C); ^1^H NMR:(DMSO-d_6_, 300 MHz, tamb) δ ppm : 3.5 (s, 3H, CH_3_), 6.4 (s, 1H, Hethy); 7.13–7.45 (m,Ar–H), ^13^C (DMSO-d_6_, 75 MHz, tamb) δ ppm : 157.2 (C_2_); 112.2 (C_3_); 144.6 (C_4_); 140.6 (Cethy); 122.1–151.2 (Carom).

### 2-(4-hydroxy-2-oxochroman-3-yl) 4-(p-methoxy)-5H-chromeno[4,3-b]pyridin-5-one 3c

Solid (Yield 80 %), mp = 234°C; IR ν cm^–1^: 1714 (C-O-C), 1683 (C=O), 1608 (C=N), 3230 (OH); ^1^H NMR:(DMSO-d_6_, 300 MHz, tamb) δ ppm: 5,47(s,1H,CH); 11,78(s,1H,OH); 6.50-8.50(m,12H, Harom), ^13^C (DMSO-d_6_, 75 MHz, tamb) δ ppm: 55.8(OCH_3_); 91,6 (C_3_);144,6(CH);115,6-134,2(Carom); 161,9(C_2_); 165,2(C_4_); 167,5(C_1′_).

### 2-(4-hydroxy-2-oxochroman-3-yl) 4-(p-methyl)-5H-chromeno[4,3-b]pyridin-5-one 3d

Solid (Yield 75 %), mp = 220°C; IR ν cm^–1^:1714 (C-O-C), 1668 (C=O), 1612 (C=N), 3192 (OH); ^1^H NMR:(DMSO-d_6_, 300 MHz, tamb) δ ppm: 2.42(s,3H,CH_3_); 5,43(s,1H,CH); 11,79(s,1H,OH); 7,26-8,02(m,12H,Harom); ^13^C (DMSO-d_6_, 75 MHz, tamb) δ ppm: 22,4(OCH_3_); 91,5 (C_3_); 115,6-133,3(Carom); 161,8(C_2_); 165,2(C_4_); 167,5(C_1′_).

### 2-(4-hydroxy-2-oxochroman-3-yl) 4-(3.4.5-trimethoxy)-5H-chromeno[4,3-b]pyridin-5-one 3e

Solid (Yield 85 %), mp = 252°C; IR ν cm^–1^: 1712 (C-O-C), 1683 (C=O), 1606 (C=N), 3230 (OH); ^1^H NMR (DMSO-d_6_, 300 MHz, tamb) δ ppm: 3,58 (s,3H, OCH_3_); 3,78 (s,3H, OCH_3_) 5,88(s,1H,CH); 6,98-8,24(m,12H, Harom), ^13^C (DMSO-d_6_, 75 MHz, tamb) δ ppm: 55,7(OCH_3_); 55,8(OCH_3_); 91,7 (C_3_); 114,6-133,3(Carom); 144,5 (C_4′_); 162,5(C_2_); 165,1(C_4_); 167,3(C_1′_).

### Antibacterial activity

Bacterial strains, media and growth conditions, Agar well diffusion method, Minimum inhibitory concentration (MIC) were done according to our work [[52], [53]].

### Antioxidant activity

DPPH radical scavenging assay and Hydroxyl radical scavenging assay were done according to our previous work [[54], [55]]

Anti-inflammatory activity was done according to our previous work [[56], [57]].

## Data availability

The authors declare that all generated and analyzed data are included in this article.

## Conflict of interest

The authors declare that we have no conflict of interest.

## Informed consent

All authors gave their consent for the publishing of the manuscript.

## CRediT authorship contribution statement

**Donia Bensalah**: Data curation. **Nasser Amri**: Writing – original draft, Formal analysis, Writing – review & editing. **Yousef E. Mukhrish**: Writing – review & editing. **Waleed S. Koko**: Validation. **Naceur Hamdi**: Writing – original draft, Data curation, Writing – review & editing.

## Declaration of Competing Interest

The authors declare that they have no known competing financial interests or personal relationships that could have appeared to influence the work reported in this paper.

## Data Availability

Data will be made available on request.

## References

[1] Viegas-Junior C., Danuello A., da Silva Bolzani V., Barreiro E.J., Fraga C.A. (2007). Curr. Med. Chem..

[2] Sandhu S., Bansal Y., Silakari O., Bansal G. (2014). Bioorg. Med. Chem..

[3] Gediya L.K., Njar V.C. (2009). Expert Opin. Drug Discov..

[4] Dasari B., Jimmidi R., Arya P. (2015). Eur. J. Med. Chem..

[5] Chen X., Decker M. (2013). Curr. Med. Chem..

[6] Decker M. (2011). Curr. Med. Chem..

[7] Grigor'ev I.A., Tkacheva N.I., Morozov S.V. (2014). Curr. Med. Chem..

[8] Sahu N.K., Balbhadra S.S., Choudhary J., Kohli D.V. (2012). Curr. Med. Chem..

[9] Batovska D.I., Todorova I.T. (2010). Curr. Clin. Pharmacol..

[10] Venugopala K.N., Rashmi V., Odhav B. (2013). BioMed Res. Int..

[11] Wu L., Wang X., Xu W., Farzaneh F., Xu R. (2009). Curr. Med. Chem..

[12] Singh P., Anand A., Kumar V. (2014). Eur. J. Med. Chem..

[13] Sashidhara K.V., Kumar A., Kumar M., Sarkar J., Sinha S. (2010). Bioorg. Med. Chem. Lett..

[14] Pingaew R., Saekee A., Mandi P., Nantasenamat C., Prachayasittikul S., Ruchirawat S., Prachayasittikul V. (2014). Eur. J. Med. Chem..

[15] Vazquez-Rodriguez S., Lama Lopez R., Matos M.J., Armesto-Quintas G., Serra S., Uriarte E., Santana L., Borges F., Munoz Crego A., Santos Y. (2015). Bioorg. Med. Chem..

[16] Xue Y., An L., Zheng Y., Zhang L., Gong X., Qian Y., Liu Y. (2012). Comput. Theor. Chem..

[17] Foroumadi A., Emami S., Sorkhi M., Nakhjiri M., Nazarian Z., Heydari S., Ardestani S.K., Poorrajab F., Shafiee A. (2010). Chem. Biol. Drug Des..

[18] Gan X., Wang Y., Hu D., Design SB. (2017). Chin. J. Chem..

[19] Han X., Peng B., Xiao B.B., Cao S.L., Yang C.R., Wang W.Z., Wang F.C., Li H.Y., Yuan X.L., Shi R., Liao J., Wang H., Li J., Xu X. (2019). Eur. J. Med. Chem..

[20] Borges F., Roleira F., Milhazes N., Uriarte E., Santana L. (2009). Front. Med. Chem..

[21] Matos M.J., Vi∼na D., Picciau C., Orallo F., Santana L., Uriarte E. (2009). Bioorg. Med. Chem. Lett..

[22] Shen Q., Peng Q., Shao J., Liu X., Huang Z., Pu X., Ma L., Li Y.M., Chan A.S.C., Gu L. (2005). Eur. J. Med. Chem..

[23] Manojkumar P., Ravi T.K., Subbuchettiar G. (2009). Acta Pharm..

[24] Ito C., Itoigawa M., Onoda S., Hosokawa A., Ruangrungsi N., Okuda T., Tokuda H., Nishino H., Furukawa H. (2005). Phytochemistry.

[25] Kostova I. (2006). Curr. HIV Res..

[26] Bhavsar D., Trivedi J., Parekh S., Savant M., Thakrar S., Bavishi A.R, Vala H., Lunagariya J., Parm L. Paresh M., Loddo R., Shah A. (2011). Bioorg. Med. Chem. Lett..

[27] Melagraki G., Afantitis A., Igglessi-Markopoulou O., Detsi A., Koufaki M., Kontogiorgis C., Hadjipavlou-Litina D.J. (2009). Eur. J. Med. Chem..

[28] Symeonidis T., Fylaktakidou K.C., Hadjipavlou-Litina D.J., Litinas K.E. (2009). Eur. J. Med. Chem..

[29] Manojkumar P., Ravi T.K., Gopalakrishnan S. (2009). Eur.J. Med. Chem..

[30] Rodriguez S.A., Nazareno M.A., Baumgartner M.T. (2011). Bioorg. Med. Chem..

[31] Kostova I., Bhatia S., Grigorov P., Balkansky S., Parmar V.S., Prasad K., Saso L. (2011). Curr. Med. Chem..

[32] Nordberg J., Arn´er E.S. (2001). Free Radical Biol. Med..

[33] Pelicano H., Carney D., Huang P. (2004). Drug Resist. Updates.

[34] L¨u J.M., Lin P.H., Yao Q., Chen C. (2010). J. Cell. Mol. Med..

[35] Slimani I., Hamzaoui S. (2020). Lamjed Mansour, Abdel Halim Harrath, Naceur Hamdi. J. King Saud Univ. Sci..

[36] Bandgar B.P., Gawande S.S., Bodade R.G., Totre J.V., Khobragade N. (2010). Bioorg. Med. Chem..

[37] Arshad M.N., Al-Dies A.M., Asiri A.M., Khalid M., Birinji A.S., Al-Amry K.A., Braga A.A.C. (2017). J. Mol. Struct..

[38] Sashidhara K.V., Kumar M., Modukuri R.K., Sonkar R., Bhatia G., Khanna A.K., Rai S., Shukla R. (2011). Bioorg. Med. Chem. Lett..

[39] Trivedi J.C., Bariwal J.B., Upadhyay K.D., Naliapara Y.T., Joshi S.K., Pannecouque C.C., Clercq E.D., Shah A.K. (2007). Tetrahedron Lett..

[40] Jagtap A.R., Satam V.S., Rajule R.N., Kanetkar V.R. (2011). Dyes Pigm..

[41] Shan Y.Y., Liu Z.Q., Cao D.X., Liu G.Q., Guan R.F., Sun N., Wang C., Wang K.N. (2015). Sens. Actuators B.

[42] García-Beltrán O., González C., Pérez E.G., Cassels B.K., Santos J.G., D.Millán N.M, Pavez P., Aliaga M.E. (2012). J. Phys. Org. Chem..

[43] Al-Ayed A.S., Hamdi N. (2014). Molecules.

[44] Dlala N.A., Bouazizi Y., Ghalla,1 H., Hamdi N. (2021). J. Chem. Volume.

[45] Dholakia V.N., Parekh M.G., Trivedi N.K. (1968). Aust. J. Chem..

[46] Hamdi M., Reine S., Patrick G., Vincent (1993). J. Hetercycl. Chem..

[47] Hantschmann A., Liebigs M., Steinfuhrer T., Weissenfels M. (1992). Ann. Chem..

[48] Patel N.B., Khan I.H., Rajani S.D. (2010). Eur. J. Med. Chem..

[49] Mosmann T. (1983). Rapid colorimetric assay for cellular growth and survival: application to proliferation and cytotoxicity assays. J. Immunol. Methods..

[50] Gangadevi V., Muthumary J. (2007). Preliminary studies on cytotoxic effect of fungal taxol on cancer cell lines. Afr. J. Biotechnol..

[51] Jelali H., Al Nasr I.S., Koko W.S., Khan T.A., Deniau E., Sauthier M., Alresheedi F., Hamdi N. (2021). J. Heterocycl. Chem..

[52] Guven K., Yucel E., Cetintas F. (2006). Antimicrobial activities of fruits of Crataegus and Pyrus species. Pharm. Biol..

[53] Sellem I., Kaaniche K., Chakchouk-Mtibaa A., Mellouli L. (2016). Anti-oxidant, antimicrobial and anti-acetylcholinesterase activities of organic extracts from aerial parts of three Tunisian plants and correlation with polyphenols and flavonoids contents. Bangladesh J. Pharmacol..

[54] Kirby A.J., Schmidt R.J. (1997). The antioxidant activity of Chinese herbs for eczema and of placebo herbs. J. Ethnopharmacol..

[55] Re P., Proteggente R., Pannala N., Yang A., Rice-Evans C.M.A. (1999). Anti-oxidant activity applying an improved ABTS radical cation decolorization assay. Free Radical Biol. Med..

[56] Yaras_ır M.N., Kandaz M., Senkal B.F., Koca A., Salih B. (2007). Polyhedron.

[57] Shinde U.A., Kulkarni K.R., Phadke A.S., Nair A.M., Mungantiwar D.V.J., Saraf M.N (1999). Ind. J. Exp. Biol..

